# Time trends in HIV-1 transmitted drug resistance mutation frequency in Poland

**DOI:** 10.7448/IAS.17.4.19753

**Published:** 2014-11-02

**Authors:** Milosz Parczewski, Magdalena Witak-Jedra, Katarzyna Maciejewska, Monika Bociaga-Jasik, Pawel Skwara, Aleksander Garlicki, Anna Grzeszczuk, Magdalena Rogalska, Maria Jankowska, Malgorzata Lemanska, Maria Hlebowicz, Grazyna Baralkiewicz, Iwona Mozer-Lisewska, Renata Mazurek, Wladyslaw Lojewski, Edyta Grabczewska, Anita Olczak, Elzbieta Jablonowska, Weronika Rymer, Aleksandra Szymczak, Bartosz Szetela, Jacek Gasiorowski, Brygida Knysz, Anna Urbanska, Magdalena Leszczyszyn-Pynka

**Affiliations:** 1 Department of Infectious, Tropical Diseases, Pomeranian Medical University in Szczecin, Szczecin, Poland; 2 Department of Infectious Diseases, Jagiellonian University Medical College, Kraków, Poland; 3 Department of Infectious Diseases and Hepatology, Medical University of Bialystok, Bialystok, Poland; 4 Department of Infectious Diseases, Medical University of Gdañsk, Gdañsk, Poland; 5 Department of Infectious Diseases, J. Strus Hospital, Poznañ, Poland; 6 Department of Infectious Diseases, Poznan University of Medical Sciences, Poznan, Poland; 7 Department of Infectious Diseases, Regional Hospital in Zielona Gora, Zielona Gora, Poland; 8 Department of Infectious Diseases and Hepatology, Nicolaus Copernicus University Collegium Medicum, Bydgoszcz, Poland; 9 Department of Infectious Diseases and Hepatology, Medical University of Lódz, Lódz, Poland; 10 Department of Infectious Diseases, Hepatology, Wroclaw Medical University, Wroclaw, Poland

## Abstract

**Introduction:**

In Poland, the HIV epidemic has shifted recently from being predominantly related to injection drug use (IDU) to being driven by transmissions among men-who-have-sex-with-men (MSM). The number of new HIV cases has increased in the recent years, while no current data on the transmitted drug resistance associated mutations (tDRM) frequency trend over time are available from 2010. In this study, we analyze the temporal trends in the spread of tDRM from 2008 to 2013.

**Materials and Methods:**

Partial pol sequences from 833 antiretroviral treatment-naive individuals of European descent (Polish origin) linked to care in 9 of 17 Polish HIV treatment centres were analyzed. Drug resistance interpretation was performed according to WHO surveillance recommendations, subtyping with REGA genotyping 2.0 tool. Time trends were examined for the frequency of t-DRM across subtypes and transmission groups using logistic regression (R statistical platform, v. 3.1.0).

**Results:**

Frequency of tDRM proved stable over time, with mutation frequency change from 11.3% in 2008 to 8.3% in 2013 [OR: 0.91 (95% CI 0.80–1,05), p=0.202] (Figure 1a). Also, no significant differences over time were noted for the subtype B (decrease from 8.4% 2008 to 6.2% in 2013 [OR: 0.94 (95% CI 0.79–1.11), p=0.45] and across non-B variants [change from 22.6% 2008 to 23.1% in 2013, OR: 0.94 (95% CI 0.75–1.19), p=0.62]. When patient groups were stratified according to transmission route, in MSM there was a trend for a NNRTI t-DRM decrease (from 6.8% 2008 to 1% in 2013, OR: 0.61 (95% CI 0.34–1.02), p=0.0655, slope −0.74%/year) (Figure 1b), related to the subtype B infected MSM (decrease from 7% 2008 to 1% in 2013, OR: 0.61 (95% CI 0.34–1.03), p=0.0662, slope −0.75%/year). Overall tDRM frequency decrease was also noted for the heterosexually infected patients [from 17.6% 2008 to 10.3% in 2013, OR: 0.83 (95% CI 0.67–1.02, p=0.077, slope −2.041%/year)] but did not associate with drug class (Figure 1c). In IDUs, the trends in t-DRM frequency were not significant over time (change from 1.9% in 2008 to 0 in 2013 [OR:1.24 (95% CI 0.73–2.26), p=0.4)].

**Conclusions:**

The frequency of t-DRM in Poland is generally stable over time. Decrease in the overall tDRM frequency in heterosexual infected cases and NNRTI resistance in subtype B infected MSM may be related to the higher treatment efficacy of current cART.

**Figure 1:**
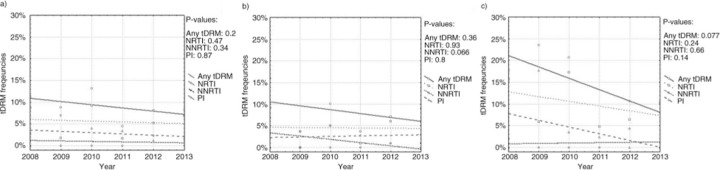
Trends in tDRM prevalence over time. (a) trends for the entire study group, (b) trends for MSM infected patients, (c) trends in for heterosexual transmissions.

